# Combining Protein Ligation Systems to Expand the Functionality of Semi-Synthetic Outer Membrane Vesicle Nanoparticles

**DOI:** 10.3389/fmicb.2020.00890

**Published:** 2020-05-12

**Authors:** H. Bart van den Berg van Saparoea, Diane Houben, Coen Kuijl, Joen Luirink, Wouter S. P. Jong

**Affiliations:** ^1^Abera Bioscience AB, Solna, Sweden; ^2^Department of Molecular Microbiology, Amsterdam Institute of Molecular and Life Sciences, Vrije Universiteit Amsterdam, Amsterdam, Netherlands; ^3^Medical Microbiology and Infection Control, Amsterdam Institute of Infection & Immunity, Amsterdam UMC, Vrije Universiteit Amsterdam, Amsterdam, Netherlands

**Keywords:** autodisplay, protein display, outer membrane vesicle, vaccine, protein ligation, Spy, Snoop, nanoparticle

## Abstract

Bacterial outer membrane vesicles (OMVs) attract increasing interest as immunostimulatory nanoparticles for the development of vaccines and therapeutic agents. We previously engineered the autotransporter protein Hemoglobin protease (Hbp) into a surface display carrier that can be expressed to high density on the surface of *Salmonella* OMVs. Moreover, we implemented Tag-Catcher protein ligation technology, to obtain dense display of single heterologous antigens and nanobodies on the OMVs through coupling to the distal end of the Hbp passenger domain. Here, we aimed to further expand the versatility of the Hbp platform by enabling the coupling of heterologous proteins to internal sites of the Hbp passenger. Inserted SpyTags were shown to be accessible at the *Salmonella* OMV surface and to efficiently couple SpyCatcher-equipped fusion proteins. Next, we combined distally placed SnoopCatcher or SnoopTag sequences with internal SpyTags in a single Hbp molecule. This allowed the coupling of two heterologous proteins to a single Hbp carrier molecule without obvious steric hindrance effects. Since coupling occurs to Hbp that is already exposed on the OMVs, there are no limitations to the size and complexity of the partner proteins. In conclusion, we constructed a versatile modular platform for the development of bivalent recombinant OMV-based vaccines and therapeutics.

## Introduction

Outer membrane vesicles (OMVs) are nanoparticles (20–100 nm) that shed from the outer membrane of Gram-negative bacteria in a natural continuous process. They are non-replicating and non-invasive, hence safe, but still contain the immunostimulatory properties that are a facsimile of the parental cells (Alaniz et al., 2007). This makes OMVs an attractive platform for the development of recombinant vaccines (Bitto and Kaparakis-Liaskos, 2017; Gnopo et al., 2017). One effective strategy involves the display of heterologous antigens on the surface of OMVs for which various concepts have been described (Gerritzen et al., 2017).

**FIGURE 1:**
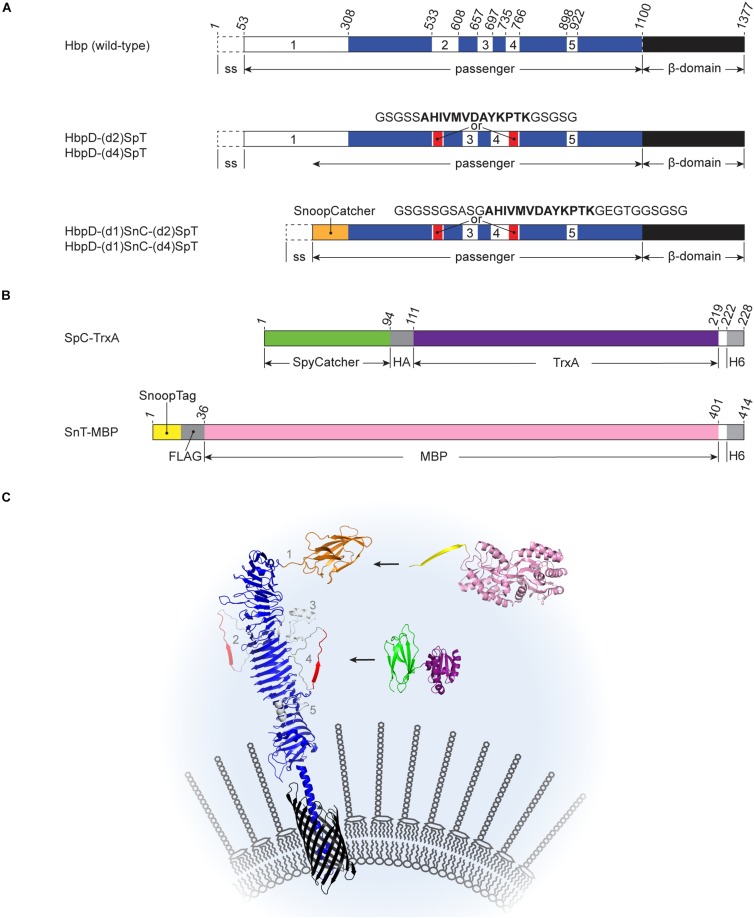
Schematic representations of Hbp derivatives and recombinant proteins used for Spy- and Snoop-based coupling at the surface of OMVs **(A)** Hbp fusions. Wild-type Hbp is synthesized with an *N*-terminal signal sequence (ss) that is cleaved off after translocation across the inner membrane. The *C*-terminal β-domain (black) integrates into the outer membrane, facilitating translocation of the passenger domain. After translocation, autocatalytic cleavage separates the passenger and the β-domain (after Asn1100). The passenger domain contains five subdomains (white, numbered 1 to 5) protruding from a β-helical stem structure (blue). The derived HbpD-(d2)SpT display platform lacks the autocatalytic cleavage site and contains a SpyTag instead of domain 2. In HbpD-(d4)SpT the SpyTag is integrated in domain 4. HbpD-(d1)SnC-(d2)SpT and HbpD-(d1)SnC-(d4)SpT are further derivatives in which domain 1 has been replaced by SnoopCatcher and the linkers surrounding the SpyTag have been extended. **(B)** Examples of Catcher- and Tag-fused model proteins. HA, HA-tag; H6, hexa histidine-tag; and FLAG, FLAG-tag. **(C)** Cartoon of Hbp-mediated Spy-and Snoop-ligation to the surface of outer membrane vesicles. HbpD-(d1)SnT-(d2 or d4)SpT is embedded in the membrane of an outer membrane vesicle. The *N*-terminal SnoopTag is available for ligation with SnC-MBP (SnC, orange; MBP, pink) and the internal SpyTag is available for ligation with SpC-TrxA (SpC, green; TrxA, deeppurple). Positions of subdomains 1–5 as present in the native Hbp passenger are indicated. The protein structure cartoons were assembled from PBD files 1WXR, 3AEH, 4MLI, 2WW8, 5HR3, and 1LLS using PyMOL.

We have previously engineered a protein display system based on the bacterial autotransporter Hemoglobin protease (Hbp; Daleke-Schermerhorn et al., 2014) to allow surface decoration of bacterial cells and derived OMVs with recombinant proteins. To cross the complex Gram-negative bacterial cell envelope, autotransporters are organized in three domains (Pohlner et al., 1987; Figure 1A): (i) an *N*-terminal signal peptide that targets the protein to the Sec translocon for translocation across the inner membrane, (ii) a secreted passenger domain that carries the effector function, and (iii) a *C*-terminal β-domain that integrates into the outer membrane (OM) and facilitates translocation of the passenger from the periplasm into the extracellular space. The latter process also involves the host-derived β-barrel assembly machinery (Bam) complex (Ieva and Bernstein, 2009; Sauri et al., 2009). Following translocation, autocatalytic cleavage in the β-domain interior results in cleavage of the passenger from its β-domain and release into the extracellular environment (Barnard et al., 2007). The cleaved and released passenger domain is a long β-helical stem structure from which small loops and larger functional domains extend (Otto et al., 2005). We previously identified five subdomains (1–5; Figure 1A) that can be replaced by heterologous polypeptides without compromising Hbp expression and secretion. Furthermore, mutagenesis of residues critical for autocatalytic cleavage created an Hbp passenger that remains anchored to the OM to allow permanent exposure of fused antigens (Jong et al., 2012). This platform was successfully used to display multiple inserted antigens from *Mycobacterium tuberculosis*, *Streptococcus pneumonia*, and pathogenic *Escherichia coli* (ETEC) on the surface of OMVs derived from a hypervesiculating and genetically LPS-detoxified *Salmonella* Typhimurium strain (Jong et al., 2012; Daleke-Schermerhorn et al., 2014; Jong et al., 2014; Kuipers et al., 2015; Hays et al., 2018). These OMVs induced strong protective responses in animal models (Kuipers et al., 2017; Hays et al., 2018), underscoring the potential of recombinant antigen-decorated OMVs for vaccination.

Although Hbp tolerates integration of heterologous protein sequences, it has a limited capacity to translocate very bulky and/or complex fusion partners (Jong et al., 2007; Daleke-Schermerhorn et al., 2014; Jong et al., 2014; Kuipers et al., 2015). With the development of protein ligation systems, such as the Tag-Catcher technology that creates covalent links between proteins (Zakeri et al., 2012; Veggiani et al., 2016), the display of complex proteins on the surface of OMVs has become much easier. This so called-bacterial superglue is based on domains of streptococcal adhesins that spontaneously form intramolecular isopeptide bonds. These domains can be split into a small Tag and a larger Catcher that reconstitute and covalently bind upon mixing, even when fused to other proteins. We have recently shown that the SpyTag and the SnoopTag, as well as the SpyCatcher and the SnoopCatcher, can be incorporated into the Hbp display platform when fused to the distal end of the Hbp passenger, thereby replacing the *N*-terminal protease domain. Full-length complex antigens as well as functional nanobodies could be linked to the OMV platform (Van Den Berg et al., 2018). Moreover, the coupling procedure appeared versatile and robust, allowing fast production of experimental vaccines, and therapeutic agents through a modular plug-and-display procedure.

Here, we aimed to further expand the versatility of the Hbp display platform by incorporating a second protein ligation site. We show that heterologous proteins can be efficiently coupled to Hbp on the surface of *Salmonella* OMVs via SpyTag at an internal position of the passenger domain. Importantly, the coupling did not affect SnoopCatcher-SnoopTag ligation at the distal end of the same passenger. The ability to couple multiple heterologous proteins simultaneously, for instance complex antigens and/or targeting moieties like antibodies and nanobodies, is of considerable interest for the development of more effective OMV-based vaccines and biomedicines.

## Materials and Methods

### Bacterial Strains and Growth Media

*Escherichia coli* BL21(DE3) was used for the production of recombinant proteins carrying Spy or Snoop elements. This strain was grown in lysogeny broth (LB; 10 g/liter tryptone, 5 g/liter yeast extract, and 10 g/liter NaCl). *S*. Typhimurium SL3261 Δ*tolRA* Δ*msbB* (Kuipers et al., 2017) was used for the isolation of OMVs and grown in TYMC (10 g/liter tryptone, 5 g/liter yeast extract, 2 mM MgSO_4_, and 2 mM CaCl_2_).

### Construction of Plasmids

Plasmid constructs and primers used in this study are listed in Table 1 and Supplementary Table S1, respectively.

**TABLE 1:** Plasmids used in this study.

Plasmid	Protein expressed	Source
pHbpD(Δd1)-SpT	HbpD with a SpyTag replacing domain 1 (AA 53-307)	Van Den Berg et al., 2018
pHbpD(Δd1)-SpC	HbpD with a SpyCatcher replacing domain 1 (AA 53-307)	Van Den Berg et al., 2018
pHbpD-(d2)SpT 5/5	HbpD with a GSGSS-SpyTag-GSGSG sequence replacing domain 2 (AA 534-607)	This work
pHbpD-(d4)SpT 5/5	HbpD with a GSGSS-SpyTag-GSGSG sequence inserted in domain 4 (replacing AA 760-764)	This work
pHbpD-(d2)SpT 10/10	pHbpD with a GSGSSGSASG-SpyTag- GEGTGGSGSG sequence replacing domain 2 (AA 534-607)	This work
pHbpD-(d4)SpT 10/10	HbpD with a GSGSSGSASG-SpyTag- GEGTGGSGSG sequence inserted in domain 4 (replacing AA 760-764)	This work
pHbpD-(d1)SnT-(d2)SpT	pHbpD-(d2)SpT 10/10 with a SnoopTag replacing domain 1 (AA 53-307)	This work
pHbpD-(d1)SnT-(d4)SpT	pHbpD-(d4)SpT 10/10 with a SnoopTag replacing domain 1 (AA 53-307)	This work
pHbpD-(d1)SnC-(d2)SpT	pHbpD-(d2)SpT 10/10 with a SnoopCatcher replacing domain 1 (AA 53-307)	This work
pHbpD-(d1)SnC-(d4)SpT	pHbpD-(d4)SpT 10/10 with a SnoopCatcher replacing domain 1 (AA 53-307)	This work
pET28 SpC-MBP	SpyCatcher-HA-MBP-His_6_	This work
pET28 SpC-TrxA	SpyCatcher-HA-TrxA-His_6_	This work
pET28 SnC-MBP	SnoopCatcher-FLAG-MBP-His_6_	This work
pET28 SnC-TrxA	SnoopCatcher-FLAG-TrxA-His_6_	This work
pET28 SnT-MBP	SnoopTag-FLAG-MBP-His_6_	This work
pET28 SnT-TrxA	SnoopTag-FLAG-TrxA-His_6_	This work

To create display construct pHbpD-(d2)SpT 5/5, a *Sac*I/*Bam*HI compatible insert encoding SpyTag flanked by 5-amino acid spacer sequences (see Table 1) was generated through annealing the long oligonucleotides SpT S/B 5 fw and SpT S/B 5 rv. The insert was ligated into the *Sac*I/*Bam*HI sites of pHbpD(Δd2; Jong et al., 2012), yielding pHbpD-(d2)SpT. To create pHbpD-(d4)SpT 5/5, a secretory version with an intact cleavage site between Hbp passenger and β-domain was created first. To this end, the annealing product of long oligonucleotides SpyTag S/B fw and SpyTag S/B rv was ligated into the *Sac*I/*Bam*HI sites of pHbp(d4in; Jong et al., 2014), yielding pHbp-(d4)SpT 5/5. Next, the *Kpn*I/*Eco*RI segment of pHbp-(d4)SpT 5/5 was replaced by that of pHbpD(Δd1) to generate display construct pHbpD-(d4)SpT 5/5.

To create 10/10 versions of pHbpD-(d2)SpT and pHbpD-(d4)SpT, an insert encoding SpyTag flanked by 10-amino acid spacer sequences (see Table 1) was generated through annealing the long oligonucleotides SpT S/B 10 fw and SpT S/B 10 rv. The insert was cloned into the *Sac*I/*Bam*HI sites of pHbpD-(d2)SpT 5/5 and pHbpD-(d4)SpT 5/5 to replace the domain 2 or domain 4 insertions, creating pHbpD-(d2)SpT 10/10, and pHbpD-(d4)SpT 10/10, respectively.

To create pHbpD-(d1)SnT-(d2)SpT, gBlocks^®^ Gene Fragment (Integrated DNA Technologies) Hbp-SpT-d2 was obtained encoding a segment of Hbp carrying a SpyTag at the position of domain 2 (Supplementary Table S2). The fragment was cloned by Gibson assembly into the *Nde*I-*Nsi*I sites of pHbpD-(d1)SnT, yielding pHbpD-(d1)SnT-(d2)SpT. Similarly, gBlocks^®^ fragment Hbp-SpT-d4, encoding an Hbp segment carrying a SpyTag in domain 4, was obtained and cloned into the *Nsi*I-*Kpn*I sites of pHbpD-(d1)SnT and pHbpD-(d1)SnC (Van Den Berg et al., 2018) to yield pHbpD-(d1)SnT-(d4)SpT, and pHbpD-(d1)SnC-(d4)SpT, respectively. Subsequently, to replace SnoopTag by SnoopCatcher, the *Xba*I/*Bam*HI segment of pHbpD-(d1)SnC was cloned into the *Xba*I/*Bam*HI sites of pHbpD-(d1)SnT-(d2)SpT, and yielding pHbpD-(d1)SnC-(d2)SpT.

MBP and TrxA encoding constructs were created as follows: The MBP encoding sequence was amplified with a *C*-terminal His_6_-tag by PCR from pET28a SnoopTag-MBP (Addgene #72323) using the primers *Eco*RI-MBP fw and *Hin*dIII-His MBP rv. Likewise, the TrxA encoding sequence was amplified with a *C*-terminal His_6_-tag by PCR from pIBA-ssTorA/TrxA(3×; Jong et al., 2017) using the primers *Eco*RI-TrxA fw and *Hin*dIII-His TrxA rv. The products were cloned *Eco*RI-*Hin*dIII into pET28 SpC-HA-SnT-His_6_ and pET28 SnC-FLAG-SpT-His_6_ replacing the SnT-His_6_ and SpT-His_6_ part (Van Den Berg et al., 2018). This yielded pET28 SpC-MBP, pET28 SpC-TrxA, pET28 SnC-MBP, and pET28 SnC-TrxA. To create SnoopTagged versions of these plasmids, a SnT-FLAG encoding DNA fragment was generated by PCR using pET28 SnC-MBP as a template and the primers *Nco*I-SnT-FLAG fw and *Bam*HI-FLAG-SpT2 rv. The fragment was cloned into the *Nco*I-*Eco*RI sites of pET28 SnC-MBP and pET28 SnC-TrxA to replace the SnC of the constructs, yielding pET28 SnT-MBP and pET28 SnT-TrxA, respectively.

### OMV Isolation, Analysis of OMV Integrity, and HbpD Surface Display

The OMV production strain *S*. Typhimurium SL3261 Δ*tolRA* Δ*msbB* carrying one of the HbpD expression plasmids (Table 1) was grown at 30°C in TYMC supplemented with glucose (0.2%), chloramphenicol (30 μg/ml), and kanamycin (25 μg/ml). Overnight precultures were used to inoculate fresh medium to an to an optical density at 660 nm (OD_660_) of 0.07. After 7 h of incubation and reaching an OD_660_ of approximately 1.0 this culture was used to inoculate fresh medium containing 50 μM of Isopropyl β-D-1–thiogalactopyranoside (IPTG) to an OD_660_ of 0.02. Growth under these inducing conditions was continued overnight. To isolate OMVs, cells were removed by two successive centrifugation steps at 5,000 × *g*. The supernatant was passed through 0.45 μm-pore-size filters (Millipore) and centrifuged at 235,000 × *g* for 1 h to sediment the OMVs. The OMVs were finally resuspended in PBS containing 15% glycerol (1 OD unit of OMVs per μl). An amount of 1 OD unit of OMVs is derived from 1 OD_660_ unit of cells. The integrity of the OMVs and the surface display of HbpD variants on OMVs was analyzed using a Proteinase *K* accessibility assay as described previously (Daleke-Schermerhorn et al., 2014).

### Protein Ligation to HbpD on OMVs

To OMVs displaying a variant of HbpD a >4-fold molar excess of purified SpC-MBP, SpC-TrxA, SnC-MBP, SnC-TrxA, SnT-MBP, and/or SnT-TrxA was added. After 24 h of incubation at 4°C, the reaction mixtures were analyzed by SDS-PAGE and Coomassie staining.

### Analysis of Protein Content of OMVs

Protein profiles of OMV samples were analyzed using SDS-PAGE and Coomassie G-250 (BioRad) staining. Densitometric analysis on Coomassie-stained gels was carried out using a Molecular Imager GS-800 Calibrated Densitometer and ImageJ software^1^. To quantify protein ligation efficiencies, the respective densities of protein bands corresponding to ligated and non-ligated Hbp fractions were calculated after correcting for the difference in molecular mass. Quantifications were performed on samples of a representative experiment and correspond to the Coomassie-stained gels shown, where appropriate. For immunodetection of protein samples after Western blotting, monoclonal anti-FLAG M2 antibody (F3165; Sigma), and HA tag monoclonal antibody (2-2.2.14; ThermoFisher Scientific) were used.

### Purification of Recombinant Proteins for Coupling to OMVs

*Escherichia coli* BL21(DE3) cells harboring a pET28a plasmid for expression of recombinant proteins carrying Spy or Snoop elements were grown in LB containing glucose (0.2%) and kanamycin (50 μg/ml) to early log phase. Protein expression was induced by the addition of IPTG to a final concentration of 0.5 mM, and the cells were incubated for a further 2 h. After centrifugation, the cells containing pellet was resuspended in buffer A (pH7.4) containing 50 mM sodium phosphate (Na_2_HPO_4_ + NaH_2_PO_4_), 300 mM NaCl, and 125 μM phenylmethylsulfonyl fluoride (PMSF). The cells were disrupted by two passages through a One Shot cell disruptor (Constant Systems, Ltd.) at 1.2 × 10^8^ Pa. Cell debris and membranes were removed by centrifugation at 10,000 × *g* and 293,000 × *g*, respectively, at 4°C. His_6_-tagged proteins were isolated from the cleared lysate using Talon Superflow medium (GE Healthcare Life Sciences) according to the manufacturer’s instructions. Eluates were dialyzed overnight at 4°C against 500 volumes of PBS (pH 7.4). After dialysis, glycerol was added to 10%, and aliquots were stored at −80°C.

## Results

### Protein Ligation to Internal Sites of the Hbp Passenger

In previous work we have shown highly efficient Spy and Snoop-based ligation of proteins to the non-cleaved Hbp display construct (HbpD) at the surface of OMVs. Tag and Catcher sequences were positioned at the “tip” of the Hbp passenger through genetic replacement of the native subdomain 1 at the *N*-terminus of the passenger (Van Den Berg et al., 2018; Figures 1A,C). Four additional subdomains (2–5) are present in the Hbp passenger that protrude from the β-helical stem (see Figure 1C) and are also tolerant toward substitution by heterologous sequences (Jong et al., 2014). To investigate whether protein ligation can be achieved at these internal positions in the Hbp passenger, we genetically integrated SpyTag (SpT) at domain 2 (d2) and 4 (d4) (Jong et al., 2007; Sauri et al., 2012; Jong et al., 2014), both located almost halfway down the β-helical stem on opposite sides (Figure 1C). To optimize its ability to contact the SpyCatcher in this context, the SpyTag was flanked by flexible 5-amino acid Gly-Ser linkers. Given the small spatial distance of the fusion sites in the Hbp passenger structure (6 Å apart; see PDB 1WXR), we considered the possibility that these short linkers (5/5) would force the 13 amino acid SpyTag sequence into a bent conformation, inhibiting functional interaction of the SpyCatcher-SpyTag pair. Hence, longer 10-residue linkers (10/10) were also tested that allow more conformational freedom, but may be more prone to proteolysis.

**FIGURE 2:**
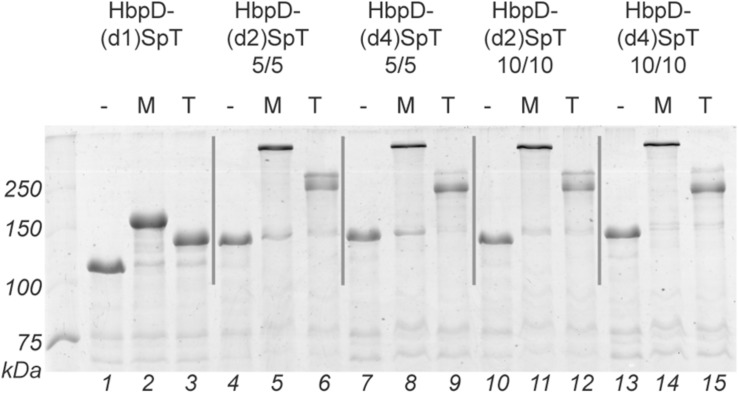
Spy ligation to the Hbp display platform containing an internal SpyTag. OMVs containing the Hbp display platform with a SpyTag at different positions (domain 1, 2, or 4) between either five amino acid long linkers (5/5) or ten amino acid long linkers (10/10) were incubated with SpC-MBP (M) or with SpC-TrxA (T). Protein ligation was analyzed by SDS-PAGE with Coomassie staining. Identical amounts of OMV material (OD_660_ units) were loaded in each lane. Below the gel image lane numbers are indicated.

OMVs were isolated from cultures of hypervesiculating *S.* Typhimurium cells expressing the generated constructs HbpD-(d2)SpT 5/5, HbpD-(d4)SpT 5/5, HbpD-(d2)SpT 10/10 or HbpD-(d4)SpT 10/10. OMVs expressing the HbpD(d1)-SpT carrying an *N*-terminally fused SpyTag (Van Den Berg et al., 2018) were produced for comparison. Analysis of the protein profiles by SDS-PAGE and Coomassie staining showed that all variants were well expressed to high densities in the OMVs, at their expected molecular mass (see Table 2), and with no apparent differences between the variants containing an internal SpyTag (Figure 2, lanes 1, 4, 7, 10, and 13).

**TABLE 2:** Spy-ligation efficiencies to HbpD on OMVs.

Displayed HbpD variant		Ligation product	
		SpC-MBP (53.5)	SpC-TrxA (24.6)
HbpD-SpT	(117)*	(171) 95%^#^	(142) 87%
HbpD-(d2)SpT 5/5	(136)	(190) 78%	(161) 84%
HbpD-(d2)SpT 10/10	(137)	(191) 83%	(162) 85%
HbpD-(d4)SpT 5/5	(144)	(198) 74%	(169) 87%
HbpD-(d4)SpT 10/10	(145)	(199) 84%	(170) 86%

*^∗^Protein molecular mass in kDa is given between brackets. ^#^Percentage of Hbp ligated with SpC-fusion protein.*

To test ligation to the HbpD-SpT fusions on the surface of the OMVs, we used two soluble model proteins of different size, the relatively small thioredoxin1 (TrxA, 12 kDa, roughly 40 × 40 × 40 Å; PDB 5HR3), and the larger maltose-binding protein (MBP, 40 kDa, roughly 45 × 65 × 70 Å; PDB 1LLS). Both were equipped with a SpyCatcher (SpC) for coupling, an HA-tag for immunodetection and a polyhistidine tag for purification (Figure 1B and Supplementary Figure S1). The OMVs were incubated overnight with purified SpC-MBP or SpC-TrxA and analyzed by SDS-PAGE (Figure 2 and Table 2). Adducts with the expected molecular mass (see Table 2) appeared upon addition of SpC-MBP and SpC-TrxA (Figure 2, lanes 2–3) at the expense of the HbpD-(d1)SpT carrier (cf. lane 1). These are specific for the presence of a SpyTag as no adducts are formed upon addition of Catchered model proteins to *Salmonella* OMVs carrying HbpD without a SpyTag (Van Den Berg et al., 2018). Densitometric scanning analysis showed 95% and 87% ligation efficiency, respectively, similar to previous ligation experiments with the same carrier (Van Den Berg et al., 2018). Efficient ligation (≥74%) to the model proteins was also seen for the HbpD constructs carrying internal SpyTags, irrespective of the length of the flanking linkers (Figure 2 and Table 2). Yet, the corresponding adducts ran with lower mobility in SDS-PAGE than expected for their molecular mass (see Table 2) and different running forms of the same adduct were observed for TrxA. This aberrant and unpredictable migration may be due to the branched nature of polypeptides upon linkage to SpyTag at the internal domain 2 and 4 positions of the Hbp passenger. Of note, immunodetection showed that these ligation products contained an HA-tag, confirming the identity of the HbpD-MBP and HbpD-TrxA adducts (Supplementary Figure S2). In conclusion, the internal domain 2 and domain 4 positions of HbpD are suitable sites for Spy-based protein ligation at the surface of OMVs.

### Simultaneous Ligation of Multiple Proteins to the Hbp Passenger

Being able to link two independent proteins at the same time would be a valuable addition to the functionality of the HbpD display platform. To achieve this, we investigated whether elements of the Spy and Snoop ligation system could be functionally combined in a single HbpD molecule. Importantly, cross-talk between these systems is absent (Veggiani et al., 2016), which should allow site-specific coupling of recombinant proteins to the Hbp platform.

Previously, we have shown efficient protein ligation using the SnoopCatcher (SnC) or the SnoopTag (SnT) at the *N*-terminal domain 1 (d1) position of Hbp (Van Den Berg et al., 2018). Likewise, to construct bivalent Spy-Snoop HbpD variants, either SnoopCatcher, or SnoopTag was placed at the domain 1 position of HbpD constructs already containing an internal SpyTag at either the position of domain 2 or 4 (Figure 1A). We chose to build on the ten-amino acid-long linker (10/10) variants only, which seemed to yield marginally more efficient protein ligation compared to the variants carrying shorter linkers (5/5; see Table 2). Moreover, future cargo proteins other than MBP or TrxA may be sterically more restricted in the coupling reaction, requiring longer linkers.

**FIGURE 3:**
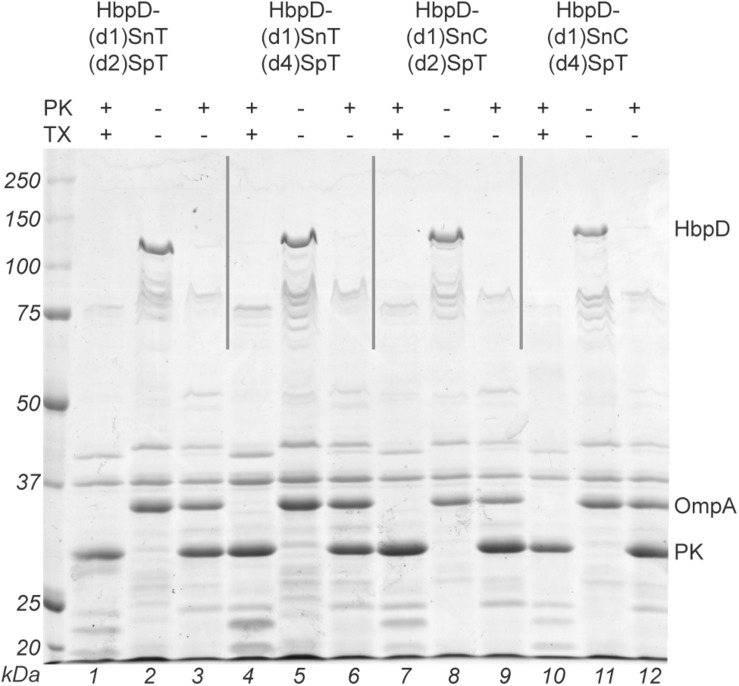
Analysis of expression and surface exposure of HbpD containing both Spy and Snoop elements. OMVs, in the absence or presence of 1% Triton X-100 (*TX*), were incubated with proteinase K (*PK*). The samples were analyzed by SDS-PAGE and Coomassie staining. Full-length HbpD fusion proteins, OmpA, and proteinase K are indicated on the right side of the panel. Below the gel image lane numbers are indicated.

Four constructs were made: HbpD-(d1)SnC-(d2)SpT, HbpD-(d1)SnC-(d4)SpT, HbpD-(d1)SnT-(d2)SpT, and HbpD-(d1)SnT-(d4)SpT (Figure 1A). OMVs from cells expressing the constructs were isolated and the fusion proteins appeared present at high density, roughly similar to the major OM protein OmpA (Figure 3, lanes 2, 5, 8, and 11). To confirm surface exposure of the passenger domains, the OMVs were treated with proteinase *K* to digest external proteins. Clearly, the HbpD derivatives were specifically degraded (Figure 3, lanes 3, 6, 9, and 12). As a control for OMV integrity, the periplasmic domain of the outer membrane protein A (OmpA) was not accessible unless the OMVs were permeabilized with Triton X-100 (Figure 3, lanes 1, 4, 7, and 10).

**FIGURE 4:**
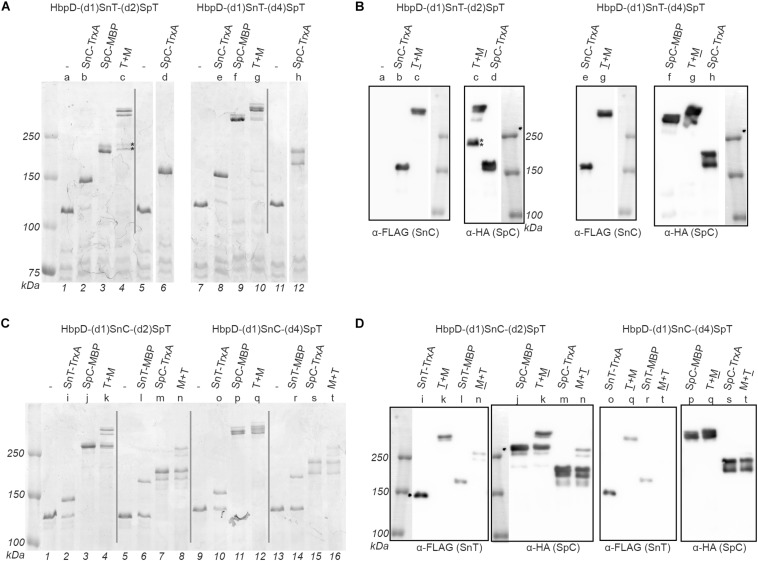
Dual coupling of proteins to the Hbp display platform. OMVs containing the Hbp display platform with either an *N*-terminal SnoopTag (SnT) or SnoopCatcher (SnC) combined with an internal SpyTag (SpT, in domain 2 or domain 4) were incubated with complementing proteins. Protein ligation was analyzed by SDS-PAGE with Coomassie staining **(A,C)** and immunodetection on Western blot **(B,D)**. Reaction mixes contained either a single complementing protein or two at the same time (T + M or M + T). Complementing proteins containing a SnT or a SnC also contained a FLAG-tag, while those with a SpyCatcher (SpC) contained an HA-tag, to identify adducts. Corresponding samples analyzed by Coomassie staining and immunodetection have been indicated with a shared number above the respective lanes. Below the gel image lane numbers are indicated. Bands in sample *c* corresponding to HbpD-(d1)SnT-(d2)SpT only coupled to SpC-MBP are indicated (*).

Model fusion proteins were produced to enable (immuno)detection of coupling to the various positions in the dual Spy-Snoop HbpD constructs. In addition to the above-described HA-tagged SpC-MBP and SpC-TrxA proteins for engagement of the internal SpyTag at the domain 2 and 4 positions, we created FLAG-tagged versions of TrxA and MBP carrying SnoopCatcher (SnC-MBP and SnC-TrxA) or SnoopTag (SnT-MBP and SnT-TrxA) for coupling to the domain 1 position (Figure 1B and Supplementary Figure S1). Of note, during purification of the model fusion proteins we noticed that SnC-MBP was rather prone to aggregation, the reason for which is unclear. This protein was not further analyzed in coupling experiments. To investigate Spy and Snoop-based protein ligation, OMVs displaying one of the four Hbp variants (see Figure 3) were incubated overnight with the cognate MBP and TrxA versions (Table 3). In the cases where simultaneous coupling to both attachment sites in the HbpD passengers was studied, the Snoop- and Spy-equipped model proteins were added together in a single reaction mix. The reaction mixes were analyzed by SDS-PAGE (Figures 4A,C) and the coupling efficiencies were determined by densitometry of the Coomassie stained carriers and adducts (Table 3). The identity of the adducts, both single and double, was confirmed by immunodetection of the HA-tag and the FLAG-tag (Figures 4B,D).

**TABLE 3:** Spy- and Snoop-coupling efficiencies to HbpD displayed on OMVs.

Displayed HbpD variant	Ligation product					
	*SnC-*… *(to d1)*		*SpC-*… *(to d2/d4)*		*SnC-*… + *SpC-*…	
HbpD-(d1)SnT-(d2)SpT (112)	TrxA (139)^∗^	88%^#^	MBP (168)	96%	TrxA + MBP (193)	71%
HbpD-(d1)SnT-(d2)SpT	*(ND)*^∧^		TrxA (137)	100%	*(ND)*	
HbpD-(d1)SnT-(d4)SpT (120)	TrxA (147)	92%	MBP (173)	98%	TrxA + MBP (201)	84%
HbpD-(d1)SnT-(d4)SpT	*(ND)*		TrxA (145)	100%	*(ND)*	
	*SnT-*… *(to d1)*		*SpC-*… *(to d2/4)*		*SnT-*… + *SpC-*…	
HbpD-(d1)SnC-(d2)SpT (123)	TrxA (139)	69%	MBP (176)	99%	TrxA + MBP (192)	43%
HbpD-(d1)SnC-(d2)SpT	MBP (168)	26%	TrxA (148)	99%	MBP + TrxA (193)	24%
HbpD-(d1)SnC-(d4)SpT (131)	TrxA (147)	55%	MBP (184)	99%	TrxA + MBP (200)	28%^&^
HbpD-(d1)SnC-(d4)SpT	MBP (176)	25%	TrxA (156)	93%	MBP + TrxA (201)	26%

*^∗^Protein molecular mass in kDa is given between brackets. ^#^Percentage of Hbp ligated with SnC-, SpC-, and/or SnT-fusion protein(s). ^∧^SnC-MBP was rather prone to aggregation and not further analyzed in coupling experiments. ^&^Estimate because the single and double adducts have similar mobility in SDS-PAGE.*

Initially we focused on coupling to the individual sites. Successful Snoop-based ligation of SnC-TrxA and SnT-TrxA to the N-terminus of Hbp in the absence of a SpyCatcher protein (Figure 4A, lane 2 and 8; Figure 4C, lane 2, 6, 10, and 14) showed that the SnoopTag as well as the SnoopCatcher can be coupled efficiently at this position (up to 92 and 69%, respectively). However, SnT-MBP ligated to the SnoopCatcher at the domain 1 position with a much lower efficiency (∼25%; Figure 4C, lane 6, and 14), which is puzzling since similar fusions to SpyTag couple more efficiently (Van Den Berg et al., 2018). On the other hand, Spy ligation to the internal SpyTag was highly efficient (between 93 and 100%) both with TrxA and MBP fusion proteins. In fact, coupling appeared to proceed more efficiently than to the comparable HbpD-(d2)SpT and HbpD-(d4)SpT constructs (see Table 2). These fusions still carried the native 27 kDa protease domain 1 at the *N*-terminus (Figure 1A), which possibly interferes with coupling to SpyTags integrated further downstream in the Hbp passenger. Remarkably, SpC-MBP adducts migrated quite differently, depending on whether coupling took place to the position of domain 2 or 4 (Figure 4A, cf. lanes 3 and 9; Figure 4C, cf. lanes 3 and 9). Coupling to either of these internal positions of the Hbp passenger did result in branched structures with different arm lengths that apparently have distinct mobilities in SDS-PAGE. Yet, these products were all detected by α-HA, confirming their identity as HbpD-MBP adducts (Figures 4B,D).

Next, we analyzed whether the TrxA and MBP model proteins could be simultaneously linked to the dual Spy and Snoop sites in the same HbpD derivative. Clearly, in all tested combinations, adducts were detected by SDS-PAGE at a higher apparent molecular mass than those formed upon incubation with the corresponding single TrxA and MBP model proteins (Figures 4A,C). Moreover, immunodetection (Figures 4B,D) demonstrated the presence of both an HA-tag and FLAG-tag in the highest molecular mass adducts in all but one sample (Figure 4C, lane 16), confirming their identity as coupling products comprising HbpD and both TrxA and MBP. Interestingly, the efficiency of formation of these adducts was roughly equivalent to the product of the two individual ligation efficiencies (Figure 4 and Table 3), suggesting that ligation to the *N*-terminal Snoop elements and the internal SpyTag in Hbp did not affect each other.

In conclusion, Spy and Snoop elements can be functionally combined in single HbpD molecules and exploited for the simultaneous coupling of different heterologous proteins at the surface of OMVs.

## Discussion

We combine OMV technology with autotransporter-based display of recombinant proteins in order to create semi-synthetic, safe and immunogenic nanoparticle vectors for vaccine formulation and therapeutic purposes. To this end, we previously genetically removed TolRA in an existing attenuated (Δ*aroA*) *S.* Typhimurium strain to overproduce OMVs for easy and cost-effective harvesting (Daleke-Schermerhorn et al., 2014). Secondly, we deleted the *msbB* gene, resulting in penta-acylated lipidA to reduce the reactogenicity of OMVs while retaining the intrinsic adjuvant effect (Kuipers et al., 2017). We further engineered the autotransporter Hbp into a scaffold that allows display of multiple integrated heterologous protein sequences (Jong et al., 2012; Jong et al., 2014). Although the Hbp display platform is relatively tolerant toward genetically inserted polypeptides, increasing the number, size and structural complexity of insertions generally lowers the expression of the fused constructs at the OMV surface due to the limited capacity of translocation across the *Salmonella* cell envelope. To overcome this limitation, the Spy and Snoop protein ligation technologies (Veggiani et al., 2016) were successfully used to covalently link purified heterologous proteins to Hbp passengers that are already present on the surface of OMVs (Van Den Berg et al., 2018).

Whereas Catchers and Tags of the ligation systems were previously located at the distal domain 1 position of the Hbp passenger, we show here that Tags also function at the domain 2 and domain 4 position, roughly halfway down the β-helical stem structure. Consistent with other studies using internally placed Tags (Zhang et al., 2013; Sun et al., 2014; Gao et al., 2016), integration of the SpyTag in Hbp did not limit its potential to couple SpyCatchered model proteins. Also, the position closer to the OM surface did not appear to hinder efficient protein ligation, as almost all Hbp-(d2)SpT and HbpD-(d4)SpT copies could be coupled (Table 3). Although not tested here, we expect that these results can be extrapolated to the integration of Tag sequences of other ligation systems such as Snoop, which was shown to function under a wide range of conditions, similar to Spy (Zakeri et al., 2012; Veggiani et al., 2016). Catchers, on the other hand, are expected to be less compatible because their integration in the Hbp passenger will likely interfere with translocation across the OM (Jong et al., 2007; Daleke-Schermerhorn et al., 2014; Jong et al., 2014; Kuipers et al., 2015). Also, fusion of the Catchers to internal positions may well affect their folding into a functional conformation, more than fusion to the passenger *N*-terminus where they have full conformational freedom. Of note, this limitation can be overcome by making use of the robust tripartite variants of both Spy and Snoop in which two short ligation Tags are covalently bonded by a separate active ligase (Fierer et al., 2014; Andersson et al., 2019).

Next, we examined the simultaneous coupling of two proteins to a single display platform molecule. To have independent control over the positioning and degree of occupation of the two separate ligation sites we chose to use the SnoopTag-SnoopCatcher pair as the secondary system. In this proof-of-principle study a non-optimized protocol was used in which OMVs carrying the HbpD fusions were simply mixed with both complementing model proteins at the same time and left to incubate. The efficiencies of dual adduct formation (Table 3) were approximately equivalent to the product of the ligation efficiencies of the individual reactions. The observation that ligation to one site is not intrinsically restrictive to ligation to the second site is important in cases where both components are needed at the highest possible density on the surface of the OMVs. Moreover, it allows high densities of dual adducts in which both attachments are fixed in close proximity to each other. A potential application for this is the simultaneous engagement of different receptors, for instance to stimulate *T* cells (Wu et al., 2020). In any case, the coupling of multiple proteins to one Hbp allows for a simple and cost-efficient production process in which only one batch of OMVs needs to be produced, without the need to express different display systems in parallel, which may be tedious. It also circumvents approaches involving the simultaneous expression of multiple Hbp constructs with a single attachment site in the same OMV production host, which may lead to instability at the genetic level due to homologous recombination events.

One obvious application of our technology is the attachment of multiple different complex antigens from any source to a single OMV for multivalent vaccine production. However, antigen display can also be combined with the coupling of for instance antibodies, nanobodies or adhesins for targeting to certain tissues and immune cells to tailor immune responses. The compatibility of our OMV platform with the attachment of SpyTagged nanobodies (Van Den Berg et al., 2018) and antibodies (data not shown) indeed suggests such a dual functionality is possible, not only for prophylactic but also for therapeutic use (Bitto and Kaparakis-Liaskos, 2017; Zhang et al., 2019). In conclusion, by functionally incorporating two protein ligation sites in HbpD we created a versatile modular platform for the development of recombinant OMV-based vaccines and therapeutics.

## Data Availability Statement

All datasets generated for this study are included in the article/Supplementary Material.

## Author Contributions

HB, DH, JL, and WJ made major contributions to the conception and design of the study. HB, DH, CK, JL, and WJ contributed to the acquisition, analysis, or interpretation of the data. HB, DH, JL and WJ contributed to the writing of the manuscript.

## Conflict of Interest

HB, DH, JL, and WJ are involved in Abera Bioscience AB that aims to exploit the presented technology. The remaining author declare that the research was conducted in the absence of any commercial or financial relationships that could be construed as a potential conflict of interest.

## Footnotes

1 http://imagej.nih.gov/ij/
