# Supplementary material for: Combining Protein Ligation Systems to Expand the Functionality of Semi-Synthetic Outer Membrane Vesicle Nanoparticles
Source: Front Microbiol. 2020 May 12;11:890. doi: 10.3389/fmicb.2020.00890 (PMC7235339; doi:10.3389/fmicb.2020.00890)
Supplement: Supplementary file 1 [file Table_1.PDF]

frontiers

TABLE S1 | Primers used in this study

Primer	Sequence (5' → 3')
SpT S/B 5 fw	ccgcccacatcgtgatggtggacgcctacaagccgacgaagg
SpT S/B 5 rv	gatcccttcgtcggcttgtaggcgtccaccatcacgatgtgggcggagct
SpT S/B 10 fw	ccggctcggctagcggtgcccacatcgtgatggtggacgcctacaagccgacgaagggtgagg gaaccggcg
SpT S/B 10 rv	gatccgccggttccctcacccttcgtcggcttgtaggcgtccaccatcacgatgtgggcaccgctag ccgagccggagct
EcoRI MBP fw	gatcgaattcaaaatcgaagaaggtaaactgg
HindIII His MBP rv	atttaagcttttagtgatgatgatgatgatggccggtacccgagctcgaattagtctgc
EcoRI-TrxA fw	gatcgaattctctgataaaattattcacctgac
HindIII His TrxA rv	atttaagcttttagtgatgatgatgatgatggccggtacccgccaggttagcgtc
NcoI-SnT-FLAG fw	actgccatgggcaaactgggcgatattgaatttattaaagtgaacaaaggatccggtagcggcgg tgactataag
BamHI-FLAG-SpT2 rv	agtcgaattccccgccgctaccgcctttatc
