# Supplementary material for: Combining Protein Ligation Systems to Expand the Functionality of Semi-Synthetic Outer Membrane Vesicle Nanoparticles
Source: Front Microbiol. 2020 May 12;11:890. doi: 10.3389/fmicb.2020.00890 (PMC7235339; doi:10.3389/fmicb.2020.00890)
Supplement: Supplementary file 2 [file Table_2.PDF]

frontiers

TABLE S2 | Synthetic DNA fragments used for cloning

Name	Sequence (5' → 3')
Hbp-SpT-d2	gctgacacagggaaccaccacatatgccatgcacgggcagcagggaaatgacctgaatgctggt<br>aagaacctgatatttcaggggcagaatggtcagattaaccttaaggattcggtttctcaggggggcgg<br>gttccctgacgttccgtgataattacacagtaacaacctctaacggaagtacctggaccggtgccgg<br>tattgttgtggacaacggggtgtccgtaaactggcaggttaatggtgttaagggcgataacctgcata<br>aaattggtgaaggtacgctgacggtacagggtacaggtattaatgaaggtggcctgaaggtcggg<br>gacggaaaggttgtactgaaccagcaggcggacaataaaggacaggtgcaggcgttcagcagt<br>gttaatattgccagtggccggccgaccgtggtactgactgatgagcggcaggtaaatccggatacc<br>gtctcatggggatatcgtgggggcacactggatgttaatggtaacagtctgacgtttcatcagttgaag<br>gcggcagattatggtgccgtgctggcgaataacgttgataaacgggccactatcacgctggactat<br>gccggttctgggagcagtggctcgggtactagtgtgcctactatcgtgatggtggacgcctacaagc<br>gttacaagggctcgagcggttccggcagcggtagtggaaatactgcagggtatctgtttcacggac<br>aactgaaaggcaatctgaatgtggacaatcgcctgcctgaaggcgttaccggtgctctggtgatgg<br>acggagctgcggatatctccggtacattcacccaggaaaacgggcgtctgacgctgcaggggcat<br>ccggttatccatgcatacaatactcagtctgtggctgacaaactggctg
Hbp-SpT-d4	tctgacgctgcaggggcatccggttatccatgcatacaatactcagtctgtggctgacaaactggctg<br>ccagtggagaccattcggttctgactcagcctacgtcattcagtcaggaggactgggagaaccgca<br>gttttacctttgacaggctgtcactgaagaacactgattttggtcttggtcgcaatgcaacactgaacac<br>aaccatccaggcagataactccagcgtcacgctgggcgacagccgggtatttatcgacaaaaac<br>gatggccagggaacagcctttacccttgaagaaggcacatctgttgcaggttctgggagcagtggc<br>tcgggtactagtgtgcctactatcgtgatggtggacgcctacaagcgttacaagggctcgagcggttc<br>cggcagcggtagtggaaaaagtgtcttcaacggcaccgtcaacctggataatcagtcagtgctga<br>atatcaatgatatattcaatggcggaatacaggcgaacaacagtaccgtgaatatctcctcagaca<br>gtgccgttctggggaactcaacactgaccagtaccgccctgaatctgaacaagggagcaaatgct<br>ctggccagtcagagttttgtttctgacggtccagtgaatatttctgatgccaccctgagtctgaacagcc<br>gtcctgatgaggtatctcacacacttttacctgtatacgattatgccggttcattgaacctgaagggag<br>acgatgcccgcctgaacgtggggccgtacagtatgttgtcaggtaatatcaatgttcaggataaagg<br>gactgtcaccctcggaggggaaggggaactgagtcctgacctgactcttcagaatcagatgttgtac<br>agcctgtttaacgggtaccgcaatatctggagcgggagcctgaatgca
