# Supplementary material for: Combining Protein Ligation Systems to Expand the Functionality of Semi-Synthetic Outer Membrane Vesicle Nanoparticles
Source: Front Microbiol. 2020 May 12;11:890. doi: 10.3389/fmicb.2020.00890 (PMC7235339; doi:10.3389/fmicb.2020.00890)
Supplement: Supplementary file 3 [file Data_Sheet_1.PDF]

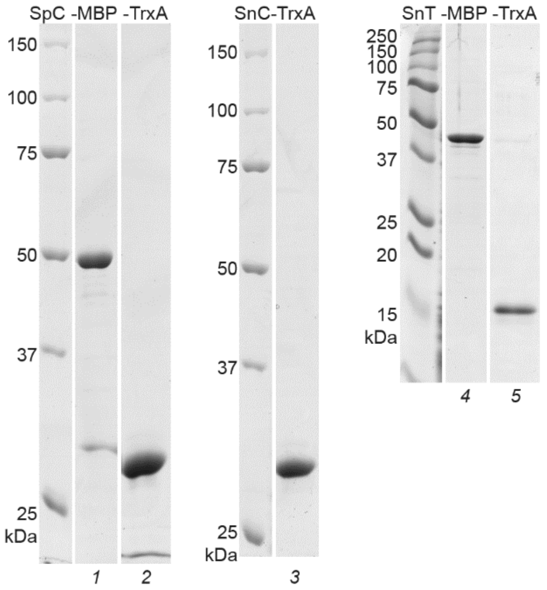

FIGURE S1 | Purified SpC-MBP, SpC-TrxA, SnC-TrxA, SnT-MBP and SnT-TrxA. Fusion proteins containing SpyCatcher (SpC), SnoopCatcher (SnC) or SnoopTag (SnT) and maltose-binding protein (MBP) or thioredoxin1 (TrxA) were purified from *E. coli* BL21 (DE3) and analyzed by SDS-PAGE and Coomassie staining. Below the gel image lane numbers are indicated.

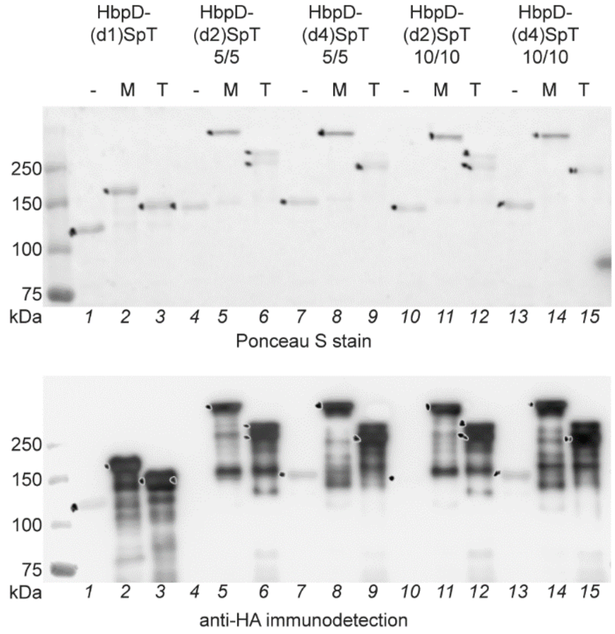

FIGURE S2 | Spy ligation to the Hbp display platform containing an internal SpyTag. OMVs containing the Hbp display platform with a SpyTag at different positions (domain 1, 2 or 4) between either five amino acid long linkers (5/5) or ten amino acid long linkers (10/10) were incubated with SpC-MBP (M) or with SpC-TrxA (T). Protein ligation was analyzed by SDS-PAGE with Coomassie staining in Figure 2. Here, the same samples were analyzed by Western blot with Ponceau S staining and anti-HA immunodetection. The immunodetection signal is overlayed with the pencil dots indicating the prominent Ponceau S stained proteins. Below the gel image lane numbers are indicated.
